# Neuronal p38α mediates synaptic and cognitive dysfunction in an Alzheimer’s mouse model by controlling β-amyloid production

**DOI:** 10.1038/srep45306

**Published:** 2017-03-31

**Authors:** Sandra Colié, Sara Sarroca, Rocío Palenzuela, Idoia Garcia, Ander Matheu, Rubén Corpas, Carlos G. Dotti, José A. Esteban, Coral Sanfeliu, Angel R. Nebreda

**Affiliations:** 1 Institute for Research in Biomedicine (IRB Barcelona), Barcelona Institute of Science and Technology, 08028 Barcelona, Spain; 2 Institut d’Investigacions Biomèdiques de Barcelona - CSIC and Institut d’Investigacions Biomèdiques August Pi i Sunyer, 08036 Barcelona, Spain; 3 Department of Molecular Neurobiology, Centro de Biología Molecular “Severo Ochoa”, CSIC - Universidad Autónoma de Madrid, 28049 Madrid, Spain; 4 School of Biosciences, Universidad Francisco de Vitoria, Pozuelo de Alarcón, 28223 Madrid, Spain; 5 Cellular Oncology Group, Biodonostia Institute, 20014 San Sebastian and Ikerbasque, Basque Foundation for Science, 48013 Bilbao, Spain; 6 ICREA, Pg. Lluís Companys 23, 08010 Barcelona, Spain.

## Abstract

Alzheimer’s disease (AD) is a neurodegenerative disorder characterized by a severe and progressive neuronal loss leading to cognitive dysfunctions. Previous reports, based on the use of chemical inhibitors, have connected the stress kinase p38α to neuroinflammation, neuronal death and synaptic dysfunction. To explore the specific role of neuronal p38α signalling in the appearance of pathological symptoms, we have generated mice that combine expression of the 5XFAD transgenes to induce AD symptoms with the downregulation of p38α only in neurons (5XFAD/p38α∆-N). We found that the neuronal-specific deletion of p38α improves the memory loss and long-term potentiation impairment induced by 5XFAD transgenes. Furthermore, 5XFAD/p38α∆-N mice display reduced amyloid-β accumulation, improved neurogenesis, and important changes in brain cytokine expression compared with 5XFAD mice. Our results implicate neuronal p38α signalling in the synaptic plasticity dysfunction and memory impairment observed in 5XFAD mice, by regulating both amyloid-β deposition in the brain and the relay of this accumulation to mount an inflammatory response, which leads to the cognitive deficits.

Alzheimer’s disease (AD) is one of the most common forms of dementia, which is characterized by a severe and progressive neuronal loss leading to cognitive dysfunctions and death^1^. The accumulation of extracellular amyloid plaques, formed by the aggregation of amyloid-β (Aβ) peptides, is a hallmark of the disease^2^. Aβ peptides derive from the cleavage of the amyloid precursor protein (APP) by a β-secretase, known as β-site APP cleaving enzyme (BACE), and γ-secretase^3^,^4^. In non-pathological conditions, instead of BACE, APP is cleaved by α-secretase (TACE or ADAM10) preventing the formation of Aβ peptide^5^. Clearance of Aβ may occur by microglial phagocytosis or by Aβ degrading enzymes like neprilysin or insulin degrading enzyme (IDE)^6^. High levels of Aβ can activate glial cells leading to the release of pro-inflammatory cytokines^7^, as well as to impaired synaptic plasticity and to cognitive deficits^8^,^9^.

p38α is a protein kinase usually activated by environmental stresses, which can be also activated by other stimuli such as growth factors and inflammatory cytokines^10^. p38α and the related family member p38β are particularly abundant in brain^11^,^12^. Several studies have reported the activation of p38 signalling during normal aging^13^ as well as in age-related neurodegenerative diseases such as AD^14^, and experiments performed *in vitro* and in mice have shown that Aβ treatment induces the activation of the p38 pathway^15^,^16^. However, the precise biological function of p38 signalling in AD is not totally understood, and a recent report provided evidence for opposite roles of different p38 family members in AD pathogenesis^17^.

Synaptic plasticity is one of the most important properties of the mammalian brain as it underlies learning and memory processes. One of the main forms of synaptic plasticity found at the excitatory synapses is the long-term potentiation (LTP), which requires the activation of postsynaptic glutamate receptors (NMDA and AMPA) and is altered in neurodegenerative diseases^18^. Chemical inhibitors of p38 have been reported to improve the Aβ-mediated LTP impairment *in vitro*^19^,^20^. Importantly, chemical inhibitors that can penetrate the central nervous system and are highly selective for p38α in kinome screens have been shown to ameliorate spatial memory deficits both in mice infused with human Aβ_1–42_ peptide and in genetic mouse models of AD^21^,^22^,^23^. Pharmacological inhibition of p38α has been also reported to reduce pro-inflammatory cytokine levels in the brain of mice infused with Aβ_1–42_ peptide^21^ and in cultured primary microglia treated with Aβ_1–42_^24^.

The above reports are consistent with the idea that p38α-mediated production of cytokines by glial cells is critical for inflammation-induced neurotoxicity, which in turn increases the risk for cognitive dysfunction. However, in addition to the role of p38α in glia, there is evidence that neuronal p38α can also mediate some AD pathological effects. For example, inhibition of p38α but not p38β has been reported to protect cortical neurons in culture from neurotoxic insults including glutamate^25^, and neuronal p38α has been implicated in the suppression of LTP induced by IL-1β in organotypic hippocampal cultures^26^. Experiments using cultured neurons and chemical inhibitors have also correlated p38 signalling with Aβ production^27^. Therefore, genetically modified mice that are deficient for p38α specifically in neurons provide a valuable tool to better understand the mechanisms regulated by p38α that impinge on Aβ deposition and the associated pathological effects.

To investigate the role of neuronal p38α in AD-like phenotypic traits *in vivo*, we have used the five familial AD mutations (5XFAD) model, which overexpresses mutant human APP and presenilin-1 (PS1), and rapidly develops severe amyloid pathology followed by synapse degeneration and cognitive deficits^28^,^29^,^30^. We found that neuronal deletion of p38α suffices to improve synaptic plasticity and memory in 5XFAD mice. Our results suggest a role for neuronal p38α in the regulation of AD pathology at multiple levels, by modulating Aβ production, neuroinflammation and neurogenesis.

## Results

### Neuronal p38α mediates cognitive dysfunctions

To investigate the role of p38α signalling in AD, we generated mice expressing CamkII-Cre and p38α-lox alleles (p38α∆-N). Downregulation of p38α did not affect the levels of p38β expressed in the brain (Supplementary Fig. S1a and S1b). Interestingly, p38α expression was higher in the hippocampus than in the cortex, whereas p38β showed the inverse expression pattern (Supplementary Fig. S1b). Adult p38α∆-N mice were healthy, showed no obvious phenotype and were also indistinguishable from the wild-type (WT) littermates in terms of non-cognitive and cognitive behaviour as illustrated by the novel object recognition test (Supplementary Fig. S1c and S1d).

We crossed the p38α∆-N mice with 5XFAD mice expressing two transgenes with AD clinical mutations, an early-onset AD mouse model^28^. Indeed, at 2–3 months of age, 5XFAD mice start to develop amyloid deposits and at 4–6 months display synaptic and memory dysfunctions^28^,^29^. The downregulation of p38α was confirmed by western blotting (Supplementary Fig. S1e). 5XFAD mice showed a lower body weight than WT mice, a difference that was maintained in the absence of neuronal p38α (Supplementary Fig. S1f). Gross brain morphology showed no significant differences between the three groups of animals (Supplementary Fig. S1g). Moreover, 5XFAD/p38α∆-N mice showed no changes in physical performance compared to 5XFAD mice, including motor activity, coordination, and grip strength (Supplementary Table S1).

**Figure 1:**
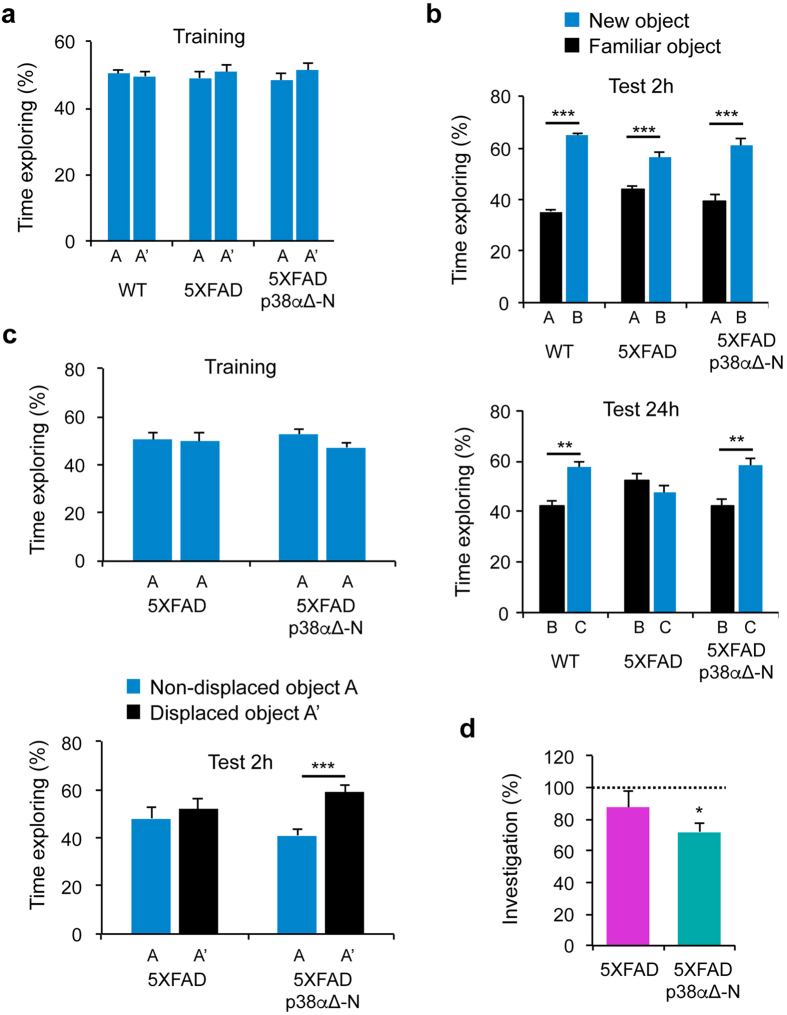
Downregulation of p38α in neurons improves the memory of 5XFAD mice. (**a,b**) The novel object recognition test was performed with 4–6 month old WT (n = 5), 5XFAD (n = 12) and 5XFAD/p38α∆-N (n = 13) male mice. The percentage of time that the mice spent exploring the novel and familiar object over the total time spent exploring both objects were calculated in the training phase (**a**), and test 2 h or test 24 h (**b**). (**c**) The object location memory test was performed with 5XFAD (n = 6) and 5XFAD/p38α∆-N (n = 9) male mice. The percentage of time that mice spent exploring the displaced and non-displaced object over the total time spent exploring both objects were calculated in the training phase and test 2 h. Results are expressed as mean ± SEM. Student *t*-test. ***p < 0.001; **p < 0.01. (**d**) The social recognition test was performed with 5XFAD (n = 5) and 5XFAD/p38α∆-N (n = 6) male mice. The percentage of time that mice spent investigating the juvenile during the test trial divided by the initial investigation time (% investigation) was calculated. Results are expressed as mean ± SEM. Dotted line indicates total lack of social recognition (hypothetical value = 100%). One sample *t*-test. *p < 0.05.

We investigated the effect of neuronal-specific downregulation of p38α in the cognitive deficits of 5XFAD mice. To evaluate short- and long-term memory, mice were subjected to the novel object recognition test. In the training phase, animals were exposed to two identical objects for 10 min (Fig. 1a). We found that regardless of the genotype, 2 h after the training phase all animals were able to distinguish the novel object from the familiar one (Fig. 1b). Interestingly, 24 h later, 5XFAD mice were clearly impaired in their ability to distinguish the novel object, whereas 5XFAD/p38α∆-N mice behaved very similar to the WT mice (Fig. 1b), suggesting that p38α downregulation improved the memory deterioration observed in 5XFAD mice. Further analysis using the object location memory test and the social recognition test confirmed that elimination of p38α from neurons ameliorates the cognitive impairment produced by 5XFAD expression (Fig. 1c and d). These results indicate that the cognitive dysfunctions induced by 5XFAD transgenes at least partially depend on neuronal p38α signalling.

### Neuronal p38α mediates synaptic plasticity alterations

**Figure 2:**
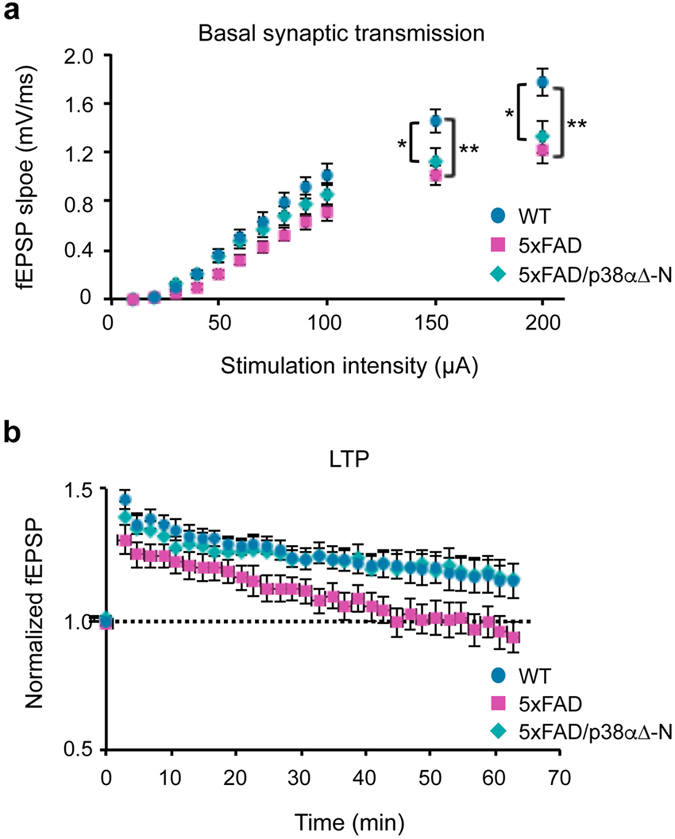
Modulation of synaptic transmission and plasticity by neuronal p38α in 5XFAD mice. (**a**) Input-output relations of field excitatory postsynaptic potentials (fEPSP slope) obtained at different stimulation intensities. Slices were obtained from the following genotypes: WT (12 slices, 4 mice), 5XFAD (14 slices, 6 mice) and 5XFAD/p38α∆-N (20 slices, 8 mice). (**b**) Time course of fEPSP responses before and after LTP induction with slices from WT, 5XFAD and 5XFAD/p38α∆-N mice. The values of fEPSP are normalized to their baseline before LTP induction. n values are as for panel (**a**). Results are expressed as mean ± SEM. Student *t*-test. ***p < 0.001; *p < 0.05.

AD mouse models are characterized by progressive synaptic depression and impaired LTP^18^. Moreover, cognitive dysfunction phenotypes usually correlate with deregulated synaptic protein expression and impaired synaptic plasticity^31^. To evaluate the implication of neuronal p38α on synaptic alterations, we used electrophysiological techniques to record CA3-to-CA1 synaptic transmission in hippocampal slices obtained from WT, 5XFAD and 5XFAD/p38α∆-N mice at 9 months of age, when synaptic deficits are already present in 5XFAD mice^29^. We found that basal synaptic transmission was reduced in 5XFAD mice, irrespective of the presence or absence of neuronal p38α (Fig. 2a). This result indicates that 5XFAD expression induces depressed transmission, as expected, but this alteration is not dependent on neuronal p38α. We then tested synaptic plasticity using a theta-burst protocol for LTP induction. WT animals displayed significant synaptic potentiation, whereas LTP was impaired in 5XFAD animals (p = 0.03 and p = 0.7, respectively). However, and in contrast with basal synaptic transmission, 5XFAD/p38α∆-N mice displayed significant LTP (p = 0.01) (Fig. 2b). These results suggest that neuronal p38α signalling contributes to the synaptic alterations that result in impaired synaptic plasticity in the 5XFAD mice.

To characterize the role of p38α in the deregulation of synaptic function, we analysed the expression of synaptophysin and PSD95, two proteins that play important roles in synapse maturation and synaptic plasticity^32^. We found that synaptophysin remained unchanged whereas PSD95 was significantly reduced in the hippocampus of 5XFAD mice compared to the WT littermates (Supplementary Fig. S2a). However, p38α downregulation did not restore the WT expression levels of PSD95. Expression of glutamate receptors GluA1 and GluN1 was also decreased in 5XFAD mice compared to WT mice (Supplementary Fig. S2b). However, the neuronal elimination of p38α had no effect in GluN1 expression, whereas GluA1 expression tent to increase but the results were not statistically significant. This decrease in postsynaptic proteins (PSD95, GluA1, GluN1) and its independence from p38α is consistent with the depression of basal transmission observed with 5XFAD and 5XFAD/p38α∆-N mice.

We also investigated the phosphorylation of GluA1 on Ser-831 and Ser-845, two residues that have been reported to potentiate AMPA receptor ion channel function^33^. We found that deletion of neuronal p38α in 5XFAD mice slightly increased Ser-831 phosphorylation, without affecting Ser-845 phosphorylation (Supplementary Fig. S2b). These results suggest that p38α could modulate the LTP response in 5XFAD mice through GluA1 phosphorylation on Ser-831.

### Neuronal p38α regulates Aβ accumulation

**Figure 3:**
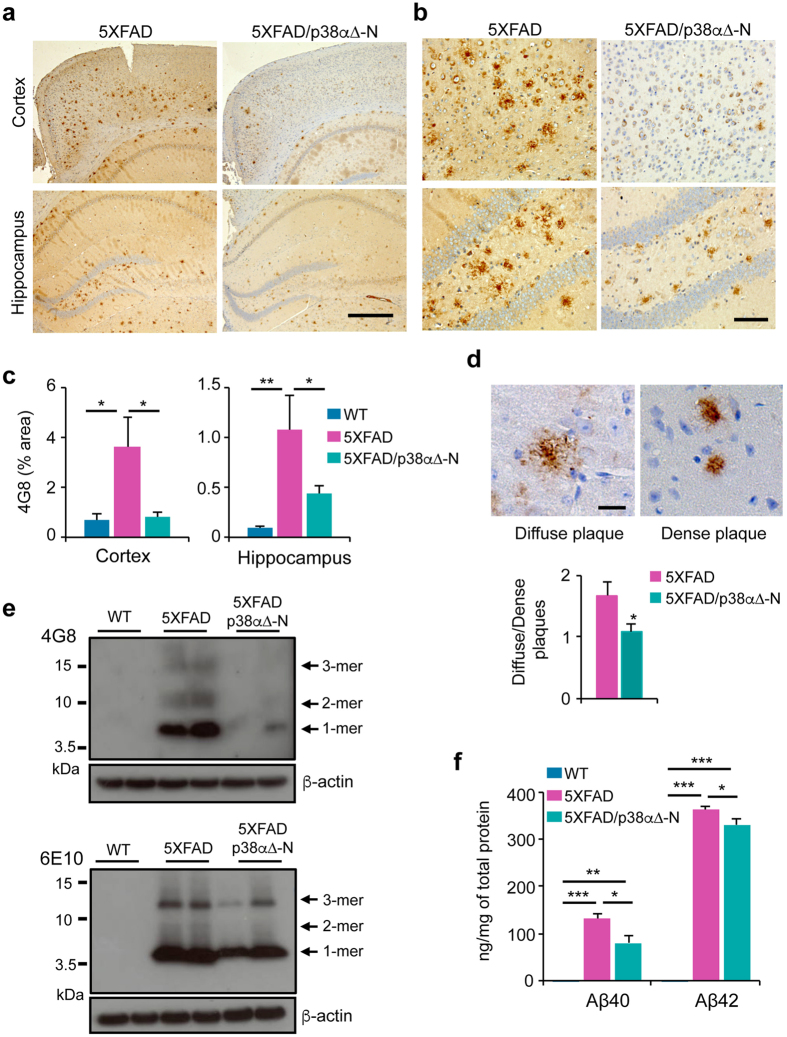
Neuronal p38α regulates amyloid-β deposition in 5XFAD mice. (**a,b**) Representative images of brain sections from 12 month old mice immunostained with the amyloid-β (Aβ) antibody 4G8. Scale bars = 500 μm (**a**) and 100 μm (**b**). (**c**) Percentage area occupied by plaques in the cortex and hippocampus was measured by Image-J. Results are expressed as mean ± SEM. (n ≥ 8). One-way ANOVA followed by Bonferroni’s multiple comparison test. **p < 0.01; *p < 0.05. (**d**) Representatives images of diffuse and dense plaques in hippocampus sections from 12-month old mice stained with the Aβ antibody 4G8. Scale bar = 20 μm. The histogram shows the ratio of the number of diffuse plaques versus dense plaques in the hippocampus of 5XFAD (n = 5) and 5XFAD/p38α∆-N (n = 6) mice. Student *t*-test. *p < 0.05. (**e**) Hippocampal lysates from 12 month old mice were analysed by western blotting with the Aβ antibodies 4G8 and 6E10. (**f**) Levels of Aβ40 and Aβ42 in cortex were measured by ELISA. Results are expressed as mean ± SEM. (n ≥ 4). Student *t*-test. ***p < 0.001; **p < 0.01; *p < 0.05.

The 5XFAD model is characterized by a rapid and strong Aβ accumulation in the brain. We looked for Aβ deposition in the brain of 12 months-old WT, 5XFAD and 5XFAD/p38α∆-N mice, by staining brain sections with a mouse anti-Aβ antibody (clone 4G8) that recognizes amino acid residues 17–24. As expected, 5XFAD mice presented a strong Aβ deposition in the cortex and hippocampus (Fig. 3a and b), and quantifications revealed that in both cases the density of Aβ staining was lower in 5XFAD/p38α∆-N mice than in 5XFAD mice (Fig. 3c).

In addition to the reduced Aβ deposits, we observed that 5XFAD/p38α∆-N mice showed similar levels of diffuse and dense plaques whereas the 5XFAD mice presented almost 60% more diffuse plaques (Fig. 3d). Since synaptic and cognitive dysfunctions are thought to be mediated by Aβ oligomers more than fibrillar plaques, we examined the level of Aβ oligomers in the brain. We found that hippocampus from 5XFAD/p38α∆-N mice displayed lower levels of Aβ oligomers than 5XFAD mice (Fig. 3e), suggesting a role for neuronal p38α signalling in the generation of Aβ oligomers in the brain *in vivo*. These results were confirmed by ELISA, which showed reduced Aβ40 and Aβ42 levels after neuronal p38α downregulation (Fig. 3f).

**Figure 4:**
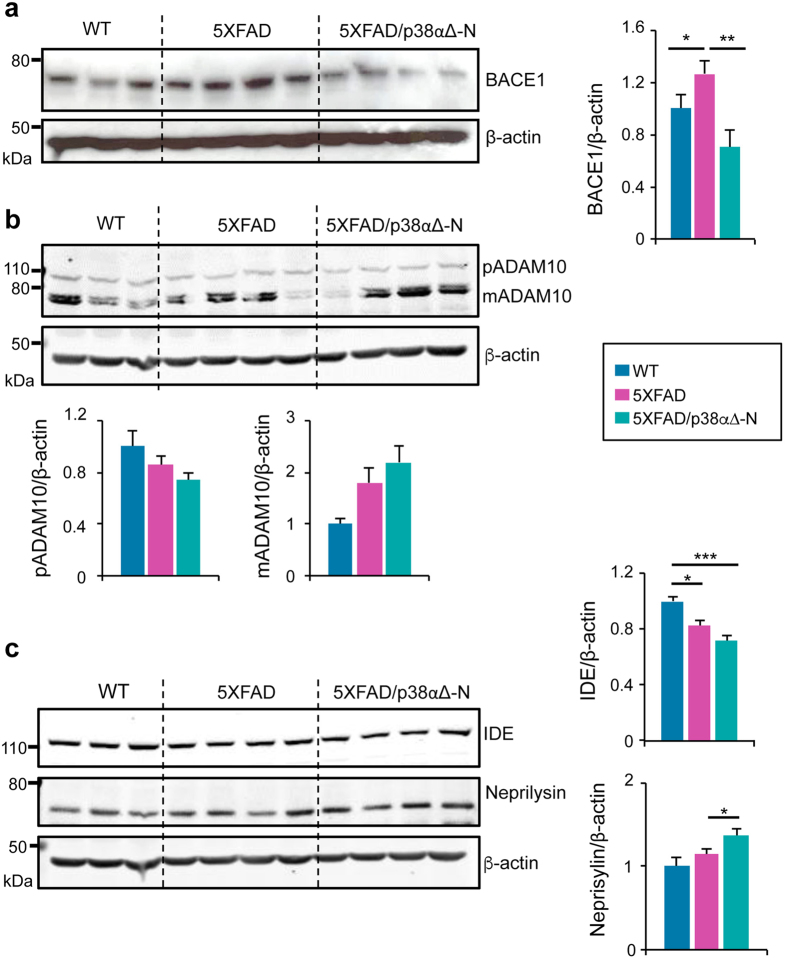
Neuronal p38α regulates BACE and neprilysin expression in 5XFAD mice. Cortical extracts from 5–6 month old WT (n = 3), 5XFAD (n = 4) and 5XFAD/p38α∆-N (n = 4) mice were analysed by western blotting (one mouse per lane) using antibodies against (**a**) BACE1, (**b**) ADAM10 to detect the pro-form (pADAMA10) and the mature form (mADAM10), and (**c**) the amyloid-β-degrading enzymes IDE and neprilysin. Histograms show the quantification normalized to β-actin. Results are expressed as mean ± SEM. Student *t*-test. ***p < 0.001; **p < 0.01; *p < 0.05.

To investigate the mechanisms by which neuronal p38α regulates Aβ levels, we evaluated the expression of the main β-secretase BACE1. We found no significant differences in BACE1 protein levels in hippocampus from 12-month old mice of the three genotypes (Supplementary Fig. S3). However, in younger animals (5–6 months old), BACE1 expression was significantly increased in 5XFAD mice compared to WT mice and this effect was completely rescued in 5XFAD/p38α∆ mice (Fig. 4a). In addition to BACE1, α-secretases, such as ADAM10, can reduce Aβ levels^5^. We observed a slight decrease in the expression of the pro-form (pADAM10) accompanied with an increase of the mature form (mADAM10) of the ADAM10 protein between 5XFAD and 5XFAD/p38α∆-N mice (Fig. 4b). Finally, since Aβ degrading enzymes produced by glial cells can also regulate Aβ levels, we analysed the expression of neprilysin and IDE in 5–6-month old mice. We observed that both 5XFAD and 5XFAD/p38α∆-N mice expressed reduced levels of IDE in comparison with WT mice. In contrast, the expression of neprilysin was higher in the cortex from 5XFAD/p38α∆-N mice than in 5XFAD mice (Fig. 4c). Taken together, these results indicate that neuronal p38α may control Aβ production by regulating the expression of BACE and neprilysin.

### Neuronal p38α modulates glial cell activation, cytokine production and neurogenesis

**Figure 5:**
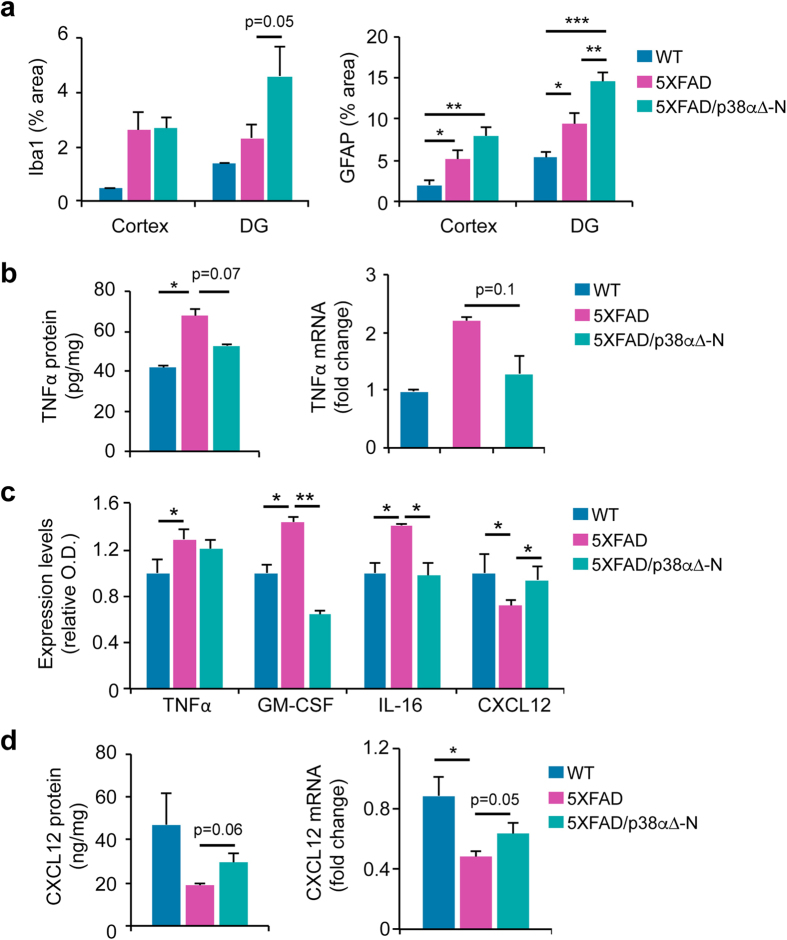
Neuronal p38α modulates astrogliosis and brain cytokine expression in 5XFAD mice. (**a**) Quantification of the intensity of Iba-1(microglia marker) and GFAP (astrocyte marker) immunoreactivity in the cortex and dentate gyrus (DG) from 5–6-month old WT (n = 3), 5XFAD (n = 6) and 5XFAD/p38α∆-N (n = 5) mice. Results are expressed as mean ± SEM. Student *t*-test. ***p < 0.001; **p < 0.01; *p < 0.05. (**b**) Cortical tissues were collected from 12 month old mice and were analysed for TNFα protein levels by ELISA and TNFα mRNAs levels by RT-PCR analysis. The experiment was performed in triplicate with a mix of cortex from four mice of each genotype. Results are expressed as mean ± SEM. (n ≥ 3). (**c**) Cortical samples were collected from 12 month old mice, and the combined protein extracts from 4 mice of each genotype were analysed using a membrane cytokine array. The arrays spots were quantified by densitometry. (**d**) CXCL12 protein levels in cortical tissues were measured by ELISA. The experiment was performed as for panel (**c**). CXCL12 mRNA expression was analysed by RT-PCR. Results are expressed as mean ± SEM (n ≥ 4). Student *t*-test. **p < 0.01; *p < 0.05.

Glial cell activation is known to participate in Aβ clearance by phagocytosis and by producing different Aβ-degrading enzymes. Since we had observed changes in Aβ-degrading enzymes and secretase expression at early times, we immunostained activated microglia and astrocytes in the cortex and the hippocampus of 5–6-month old mice (Supplementary Fig. S4). Quantifications revealed increased staining for both Iba1 and GFAP (Fig. 5a) in 5XFAD mice compared to WT mice. Interestingly, we could measure a further increase in activated microglia and astrocytes in the 5XFAD/p38α∆-N mice compared with 5XFAD mice. This data is consistent with the idea that in the early stages of the disease, the absence of neuronal p38α may facilitate the activation of glial cells to induce Aβ degradation.

Since activated microglia can produce inflammatory cytokines such as TNFα, we investigated the implication of neuronal p38α deletion in cytokine production in 5XFAD mice. We found that cortical tissues from 5XFAD mice expressed higher levels of TNFα mRNA than WT mice (Fig. 5b). Interestingly, TNFα levels were reduced in the cortex of 5XFAD/p38α∆-N mice compared with 5XFAD mice (Fig. 5b), indicating a possible contribution of TNFα to the cognitive dysfunction induced by 5XFAD transgene expression. Further analysis identified three more cytokines, whose expression in cortex was significantly altered upon neuronal downregulation of p38α in 5XFAD mice (Fig. 5c). Granulocyte macrophage colony-stimulating factor (GM-CSF) and IL-16 were both increased in 5XFAD mice compared to WT mice, and these changes were reversed in the 5XFAD/p38α∆-N mice. On the other hand, the chemokine CXCL12, also known as stromal cell-derived factor-1 (SDF-1), was reduced in the 5XFAD mice compared to WT mice, whereas 5XFAD/p38α∆-N mice showed similar levels as the WT mice. We confirmed by RT-PCR and ELISA that CXCL12 levels were decreased in the 5XFAD mice compared to the WT mice, and were partially rescued in 5XFAD/p38α∆-N mice (Fig. 5d). Thus, neuronal p38α signalling regulates the production of key cytokines in response to 5XFAD transgene expression.

**Figure 6:**
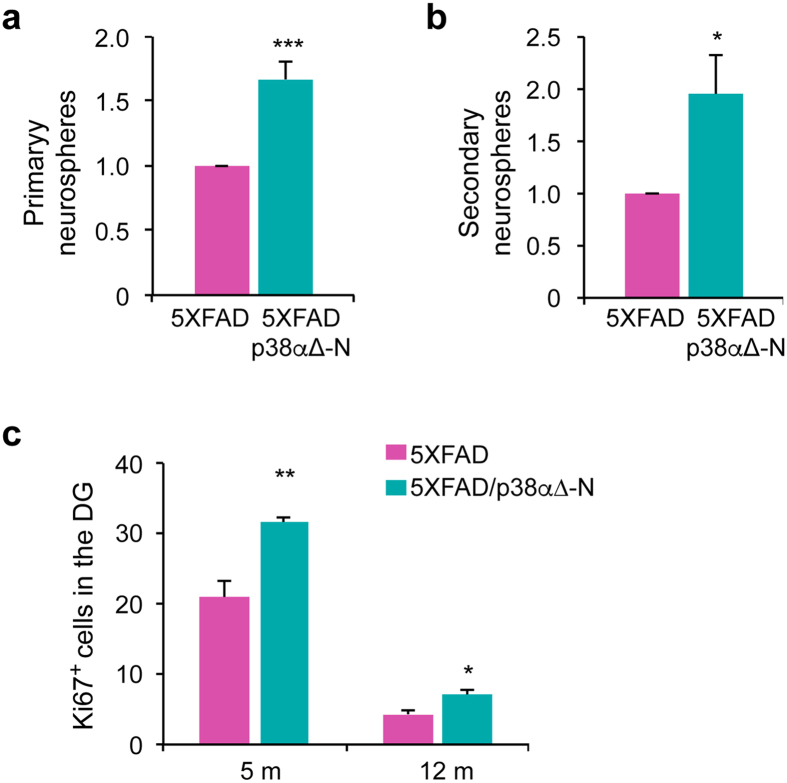
Neuronal p38α modulates neurogenesis in 5XFAD mice. (**a**,**b**) Neurosphere formation assay in the dentate gyrus (DG) from 9 month 5XFAD and 5XFAD/p38α∆-N mice. The number of neurospheres is shown relative to the 5XFAD mice. Results are expressed as mean ± SEM (n = 4). Student *t*-test. ***p < 0.001; *p < 0.05. (**c**) Quantification of Ki67^+^ cells in the DG of 5 and 12 months old mice. Results are expressed as mean ± SEM (n = 4). Student *t*-test. **p < 0.01; *p < 0.05.

There is evidence that neurogenesis is altered in the early stages of AD, which may contribute to synaptic dysfunction^34^,^35^. We found that the ability of dentate gyrus cells from 5XFAD/p38α∆-N mice to form both primary and secondary neurospheres was significantly increased in comparison with DG cells from 5XFAD mice (Fig. 6a and b). Moreover, we found an enhanced number of Ki67^+^ cells in the dentate gyrus of 5XFAD/p38αΔ-N mice compared with 5XFAD mice at both 5 and 12 months (Fig. 6c). These results suggest a role for p38α in the regulation of neural stem cells proliferation and self-renewal.

## Discussion

We have investigated the role of neuronal p38α signalling in the 5XFAD mouse model of AD. This model has been useful to study mechanisms linked to AD and to assay potential neuroprotective therapies. 5XFAD mice rapidly develop severe amyloid pathology followed by synapse degeneration and cognitive deficits^28^, with neuronal loss occurring at later stages^36^. We show that genetic deletion of p38α in neurons rescues the LTP and memory impairment of 5XFAD mice. These improvements are associated with a decrease in Aβ accumulation and reduced production of specific inflammatory cytokines.

The accumulation of Aβ in the brain is a critical event in AD pathology, and reflects an imbalance between its production and degradation. However, the cause of the imbalance is not totally understood. Studies in 5XFAD mice have contributed to establish the role of BACE1 in AD pathology. Thus, BACE1 deletion in 5XFAD mice shows neuroprotective effects, and it has been proposed that the amyloid pathway may trigger BACE1 elevation, which then drives a positive feedback loop that causes further amyloid generation^37^,^38^. Previous work has shown that chemical inhibition of stress-activated protein kinases including p38 impair BACE1 upregulation in cultured neurons exposed to 4-hydroxynonenal^27^. Moreover, a report published while we were preparing our work for submission, has shown that p38α downregulation using NEX-Cre decreases Aβ load and BACE1 expression in APP/PS1-expressing mice^39^. Our results are consistent with the idea that p38α signalling induces BACE-1 expression in adult neurons of 5XFAD mice.

We provide evidence that other mechanisms may also contribute to the reduced Aβ accumulation observed in 5XFAD/p38α∆-N mice. Thus, we detected increased expression of matured ADAM10 in 5XFAD mice with p38α-deficient neurons supporting that α-secretase-dependent cleavage of APP could avoid the cleavage by BACE1, therefore contributing to Aβ clearance. The absence of neuronal p38α could impinge on the ability of astrocytes to produce α-secretases, perhaps by regulating the secretion of soluble factors. Moreover, 5XFAD/p38α∆-N mice show increased expression of the Aβ-degrading enzyme neprilysin that correlates with astrocyte and microglial cell activation, which like neurons are known to express Aβ-degrading enzymes^6^. Of note, genetic downregulation of neprilysin increases Aβ deposition and aggravates the behavioural and neuropathological phenotype of 5XFAD mice^40^. Taken together, our results support that neuronal p38α may contribute to Aβ deposition in the brain both through the induction of BACE1 in a cell autonomous manner, and by reducing the recruitment or activation of glial cells that contribute to Aβ degradation.

Our study indicates that deletion of p38α in adult neurons suffices to rescue both LTP and memory loss in 5XFAD mice. Previous reports have shown that lowering BACE1 expression in 5XFAD mice, in addition to reducing Aβ levels, prevents LTP and memory deficits^37^,^41^,^42^. Taken together, it seems likely that p38α signalling in adult neurons regulates Aβ accumulation, which in turn induces synaptic plasticity dysfunction and cognitive deficits. However, it should be noted that p38α signalling can also contribute to Aβ-mediated LTP impairment, as shown in cortex and hippocampal slices treated with chemical inhibitors^19^,^20^. Of note, a recent report has shown that site-specific phosphorylation of Tau by p38γ reduces Aβ toxicity in AD mouse models^17^, which is the opposite effect of p38α. This emphasizes the importance of designing experiments that elucidate the function of specific p38 family members.

Inflammation plays an important role in the appearance and progression of AD, and 5XFAD mice show progressive neuroinflammation, which allowed to address different roles of microglia in the course of AD pathology^43^,^44^. Consistent with previous reports showing that systemic treatment with a brain-penetrant p38α chemical inhibitor reduces pro-inflammatory cytokine levels in the brain of mice treated with Aβ peptide^21^,^22^, we show that the increased levels of TNFα observed in 5XFAD mice are reduced in 5XFAD/p38α∆-N mice. Pro-inflammatory cytokines such as TNFα contribute to LTP inhibition, which can be mediated by p38 signalling^8^. Our results confirm the involvement of p38α signalling in LTP inhibition. This could be operating both at the level of TNFα production and by mediating the TNFα effect. Moreover, we show that impaired p38α signalling in adult neurons of 5XFAD mice also reduces GM-CSF levels. In astrocytes, p38α induces the expression of GM-CSF, which leads to the release of inflammatory cytokines and to neuronal death^45^. Our results indicate that p38α in neurons regulates GM-CSF production and the release of inflammatory cytokines such as TNFα implicated in LTP inhibition.

5XFAD/p38α∆-N mice show reduced expression of IL-16 compared with 5XFAD mice, and IL-16 is an important chemoattractant for T cell activation^46^. Little is known about the possible role of IL-16 in AD, although altered IL-16 levels have been reported in the plasma from AD patients suggesting that IL-16 may be implicated in the neuroinflammatory component of the disease^47^. Moreover, 5XFAD mice display reduced levels of CXCL12/SDF-1α, which are recovered upon neuronal p38α downregulation. CXCL12 is produced by astrocytes as well as by neurons and has been implicated in brain plasticity, but can also function as chemoattractant for lymphocytes and macrophages, and in neuro-glia communication^48^. Intracerebroventricular administration of CXCL12 reduces Aβ deposits in AD mice, which is associated with an increase of cells positive for the microglia marker Iba1, suggesting that CXCL12 recruits migroglia cells to induce Aβ clearance^49^. Since the reduced Aβ levels and increased microglia and astrocyte activation observed in 5XFAD/p38α∆-N mice correlate with partially restored CXCL12 levels, it is possible that CXCL12 downregulation by neuronal p38α contributes to Aβ accumulation. Moreover, impaired signalling by the CXCL12 receptor CXCR4 has been associated to cognitive decline^50^, suggesting that increased CXCL12 levels could also contribute to the improved memory of 5XFAD/p38α∆-N mice.

There is evidence indicating that hippocampal neurogenesis is impaired in mouse models of AD including 5XFAD mice^51^. This could be responsible, at least in part, for neuronal vulnerability as well as for the synaptic plasticity deficit and memory loss observed in AD^34^. We have found that p38α downregulation in neurons ameliorates the impaired neurogenesis of 5XFAD mice. Of note, CXCL12/CXCR4 signalling has been described to regulate neurogenesis during embryo development^52^, and in adult brain to counteract local damage^53^. Therefore, p38α signalling might contribute to the impaired neurogenesis associated with brain pathologies such as AD.

In summary, p38α downregulation in adult neurons of 5XFAD mice results in decreased Aβ accumulation, probably by a mechanism dependent on the modulation of both BACE1 and Aβ degrading enzymes. This Aβ decrease is associated with reduced levels of pro-inflammatory cytokines in brain, as well as restored LTP and improved memory response. It would be interesting to test the role of neuronal p38α in AD mouse models that do not overexpress APP^54^. Taken together, our results indicate that p38α signalling in neurons may participate in the regulation of both Aβ deposition in the brain and the deleterious effects of Aβ accumulation, further supporting the potential interest of targeting p38α for the treatment of AD.

## Methods

### Mice

To induce forebrain-specific downregulation of p38α, B6.Cg-Tg(Camk2a-cre)T29-1Stl/J mice obtained from the Jackson Laboratory (Bar Harbor, ME, USA) were bred with p38α-floxed mice in C57BL/6 background^55^,^56^. Animals for experimentation were maintained in C57BL/6 background and were obtained by crossing p38α ^(lox/lox)^ Camk2a-Cre males or females with p38α ^(lox/lox)^ mice of the opposite sex. To combine the forebrain-specific p38α deletion with the 5XFAD model, we used heterozygous 5XFAD transgenic mice purchased from The Jackson Laboratory. These mice overexpress in the brain mutant human APP(695) with the Swedish (K670N, M671L), Florida (I716V), and London (V717I) Familial Alzheimer’s Disease (FAD) mutations along with human PS1 harbouring two FAD mutations, M146L and L286V, bot transgenes driven by the mouse *Thy1* promoter. We crossed 5XFAD Tg/+ males (B6SJL background) with p38α ^(lox/lox)^ females (C57BL/6 background), and the resulting 5XFAD Tg/+p38α ^(lox/+)^ males were crossed again with p38α ^(lox/lox)^ females to obtain 5XFAD Tg/+p38α ^(lox/lox)^ mice. For experimentation, 5XFAD Tg/+p38α ^(lox/lox)^ males were crossed with p38α ^(lox/lox)^ Camk2a-Cre females to obtain 5XFAD Tg/+p38α ^(lox/lox)^ mice with or without Cre. Littermate mice that do not express the 5XFAD transgene were used as controls. We did not detect significant fertility problems in p38α ^(lox/lox)^ Camk2a-Cre mice or 5XFAD Tg/+p38α ^(lox/lox)^ Camk2a-Cre mice. Mice were genotyped for *MAPK14* (p38α), Camk2a-Cre and 5XFAD by gene-specific PCRs using tail genomic DNA. Primers and conditions are available upon request. Mice were maintained and handled according to national and European Union regulations.

### Cognitive tests

Mice were handled in compliance with the relevant national and international guidelines following protocols approved by the Animal Care Ethic Committee of Barcelona University.

#### Novel object recognition test

This test evaluates short- and long-term recognition memory involving hippocampus and cerebral cortical areas^57^,^58^. The experimental apparatus used for this test was a 90°, two-arm, 25-cm-long, 20-cm-high, and 5-cm-wide maze made of black polyvinyl chloride. A video camera hanging from the ceiling recorded the mouse movement for their posterior analysis. This test consisted of a period of habituation, an acquisition trial and two test trials (2 h and 24 h after the acquisition trial). During habituation, the mouse was allowed to freely explore the apparatus without objects, for 10 min on two consecutive days. On the acquisition trial of the next day, two identical novel objects (A + A′) were placed at the end of each arm, and the animal was allowed to explore them freely for 10 min. The mouse was then removed from the apparatus and returned to its home cage. Two h after the acquisition trial, one of the objects was replaced by a novel object with a different shape and colour (A + B), and the mouse was allowed to explore the maze for another 10 min. Twenty-four h after the acquisition trial, the mice were tested again, with a new object and an object identical to the new one in the previous trial (B + C). The time spent exploring each object was quantified from the video recordings from each trial session. Exploration of an object was defined as pointing the nose towards the object at a distance ≤1 cm and/or touching it with the nose. Turning around or sitting on an object was not considered exploration. To analyse cognitive performance in each trial, the percentage of time that mice spent exploring the novel and familiar object over the total time spent exploring both objects were calculated.

#### Object location test

This test evaluates spatial memory, a hippocampal-dependent task^58^,^59^,^60^. The experimental apparatus used for this test was an open-field box (40 cm wide × 30 cm deep × 30 cm high) made of black polyvinyl chloride with a video camera hanging from the ceiling to record the mouse movement. Identical plastic columns (10 cm in height ×5 cm in diameter) were used as objects. This test consisted of a period of habituation, an acquisition trial and a test trial. During habituation, the mice were allowed to freely explore the apparatus without objects, for 10 min on the day before the acquisition trial. On the acquisition trial, each mouse was allowed 5 min to explore two identical objects (A1 + A2) that were placed in the corners of the experimental apparatus. The mouse was then removed from the apparatus and returned to its home cage. The test trial was conducted 1 h after the acquisition trial. On the test trial, one of the objects (A2) was moved to a different location (A2′) and the other object (A1) retained in the same position (A1) as in the acquisition trial. The mouse was reintroduced into the experimental apparatus for 5 min, and the time spent exploring each object (A1 + A2′) was recorded again. Exploration of an object was defined as pointing the nose towards the object at a distance of ≤1 cm and/or touching it with the nose. Turning around or sitting on an object was not considered exploration. To analyse cognitive performance, the percentage of time that mice spent exploring the displaced and non-displaced object over the total time spent exploring both objects were calculated.

#### Social recognition test

This test evaluates social recognition memory, another hippocampal-dependent task^61^,^62^. The test consisted of a period of habituation, an initial trial and a test trial 3 h after the initial trial. During habituation, the adult male mice to be tested were placed into a clean individual cage (the same acrylic cage used for housing) and allowed to freely explore it for 15 min prior to the experimental sessions. On the initial trial, a male juvenile mouse was placed into the cage with the test mouse for 2 min. The juvenile mouse was then returned to its home cage. After 3 h, the same juvenile mouse was placed back into the test mouse’s cage for another 2 min. The time spent investigating the juvenile by the test mouse was quantified from each trial session. Social investigation behaviour includes direct contact with the juvenile, sniffing of the mouth, ears, tail, ano-genital area, and close following (within 1 cm) of the juvenile. Any aggressive encounter between animals was immediate cause for terminating the experiment and excluding data from the analysis. To measure social recognition memory, the percentage of time that mice spent investigating the juvenile during the test trial divided by the initial investigation time (% investigation) was calculated.

### Electrophysiology

Acute hippocampal slices of 400 μm-thickness were obtained from 9 month old male mice (WT, 5XFAD and 5XFAD/p38α∆-N), with a dissection solution containing 10 mM D-glucose, 4 mM KCl, 26 mM NaHCO_3_, 233.7 mM sucrose, 5 mM MgCl_2_. Slices were then maintained for 1 h at 32 °C in artificial cerebrospinal fluid (ACSF) before recordings. ACSF contained 119 mM NaCl, 2.5 mM KCl, 26 mM NaHCO_3_, 1 mM NaH_2_PO_4_, 11 mM D-glucose, 2.5 mM CaCl_2_, 1.2 mM MgCl_2_, saturated with 95% O_2_/5% CO_2_. Synaptic responses were evoked with bipolar stimulation electrodes and recorded with a low-resistance (0.2–0.8 MΩ) glass electrode filled with ACSF, placed in CA1 stratum radiatum. Field excitatory postsynaptic potentials (fEPSPs) were recorded at different stimulation intensities for each slice to generate an input-output curve. This curve was also used to set the baseline fEPSP value at ≈30% of the maximum for LTP experiments. Baseline stimulation was delivered every 15 sec (0.01 msec duration pulses) for at least 20 min before LTP induction to ensure stability of the response. LTP was induced with a theta-burst stimulation protocol: bursts of 4 pulses at 100 Hz, with the bursts repeated in 10 trains at 5 Hz, and the trains repeated 4 times separated by 15 sec.

### Brain samples

Animals were weighted and decapitated; immediately the brain was harvested and separated sagitally in two hemispheres. One hemibrain was fixed overnight by immersion in 4% paraformaldehyde (PFA) at 4 °C and embedded in paraffin. The other hemibrain was dissected on ice to obtain hippocampus and cerebral cortex and samples were stored at −80 °C for further analysis. One week after the object recognition test, half of the animals were weighted and decapitated. The brain was dissected on ice to obtain hippocampus and cerebral cortex and samples were stored at −80 °C for further analysis. The other half was anesthetized by intraperitoneal injection of ketamine (150 mg/kg) and xylazine (10 mg/kg) and then transcardially perfused with PBS followed by 4% of PFA. Afterwards, brains were dissected, post-fixed overnight in 4% PFA at 4 °C and embedded in paraffin.

### Amyloid-β ELISA

The levels of soluble amyloid-β40 and amyloid-β42 were analysed in cerebral cortex tissues using the ELISA kits from Invitrogen KHB3481 and KHB3441, respectively, following the manufacturer’s instructions.

### Cytokine array

To detect cytokine levels in the brain, cortical tissues were isolated from 4 different mice of the same genotype and prepared as described in the mouse cytokine array’s protocol (R&D systems, ARY006). Briefly, the array, which contained 40 different antibodies printed in duplicate, was incubated overnight at 4 °C with 300 μg of cortex lysate. After several washes, the array was incubated for 30 min with streptavidin-horseradish peroxidase and membranes were exposed to chemiluminescent reagent mix. Positive spots were detected by autoradiography according to the manufacturer’s instructions and quantify by Image J software.

### TNFα and CXCL12 ELISA

The levels of TNFα and CXCL12 were analysed in cerebral cortex tissue using ELISA kits from Invitrogen (KMC3011) and from R&D systems (MCX120), respectively, following the manufacturer’s instructions. Brain samples were prepared as described for the cytokine array and 200–500 μg of brain extracts were used for quantification.

### RNA extraction and real-time PCR

Total RNA from cerebral cortex and hippocampus tissues was isolated using the PureLink RNA mini kit (Ambion #12183018A) according to the manufacturer’s protocol and treated with DNase (PureLink DNAse; Invitrogen). RNA was quantified using a Nanodrop spectrophotometer. An aliquot of 1 μg of total RNA was reversed-transcribed (SuperScript II; Invitrogen #18064–014) and used as a template for real-time PCR. The reactions were performed in triplicate using 4 μl of 1/10 diluted cDNA on a Bio-Rad C1000 thermal cycler machine using SYBR Green (Bio-Rad, 48190–011). Relative quantities (∆ cycle threshold values) were obtained by normalizing against Rpl32. The primers used are listed in the Supplementary Table S2.

### Immunoblotting

Cerebral cortex and hippocampal tissues were mechanically disrupted in lysis buffer containing 20 mM Tris/HCl pH 7.4, 150 mM NaCl, 4 mM EDTA, 0.5% Triton X-100, 0.2% SDS, 20 mM NaF, 0.1 mM sodium orthovanadate, 2 mM PMSF, 1 μM microcystin, 1 mM DTT, Protease Inhibitor Cocktail Set III (Merck Millipore) and PhosStop Phosphatase inhibitor cocktail (Roche). The homogenate was incubated on ice for 15 min, sonicated and centrifuged at 4 °C for 10 min at 16,000 xg. Protein content was quantified using the Protein Assay kit (Bio-Rad) with BSA as standard, and 20–50 μg of total protein lysate were separated on SDS-PAGE and transferred to a nitrocellulose membrane. After blocking (5% non-fat milk and 1% BSA in PBS, 1 h at RT) membranes were incubated at 4 °C overnight with the primary antibodies listed in the Supplementary Table S3. After washing with PBS, membranes were incubated with Alexa Fluor 680 or 800-conjugated secondary antibodies (Molecular Probes; 1:3000) for 1 h at RT and were visualized using Odyssey Infrared Imaging System (Li-Cor, Biosciences). Band intensities were quantified by densitometric analysis using β-actin as loading control. For the detection of Aβ, protein lysates were separated in a NuPAGE^®^ Novex^®^ 4–12% Bis-Tris Gels (Invitrogen, NP0336BOX). After transfer to nitrocellulose, membranes were boiled for 5 min in PBS, blocked and incubated at 4 °C overnight with the primary antibodies. After washing with PBS, membranes were incubated with HRP conjugated anti-mouse (Dako, P0447) for 1 h at RT, and exposed to chemiluminescent reagent mix (Millipore, WBKLS0050).

### Histology and immunohistochemistry

For histological analysis, mice were anesthetized by intraperitoneal injection of ketamine (150 mg/kg) and xylazine (10 mg/kg) and then transcardially perfused with PBS followed by 4% of paraformaldehyde (PFA). Afterwards, brains were dissected, post-fixed overnight in 4% PFA at 4 °C and embedded in paraffin. Paraffin-embedded brain sections were stained with Hematoxylin/Eosin (H&E) and cresyl violet in order to analyse brain morphology. Immunostainings were performed for 1–2 h at RT using antibodies against amyloid-β 4G8 (Covance, SIG-39320, 1/50), Iba1 (Wako, 019–19741, 1:2000), GFAP (Dako, Z0334, 1:800), Ki67 (Novocastra, Ki67P-CE, 1:1000). The secondary antibodies were HRP conjugated anti-rabbit (ImmunoLogic, DPVR110HRP, RTU), anti-mouse (Dako, P0447; 1:100), anti-goat (Dako, P0449; 1:80) and anti-rat (Dako, P0450; 1:75) and were all incubated for 30–45 min at RT. Signals were visualized with DAB (3,3-diaminobenzidine), using hematoxilin as a counterstaining.

Brain pictures were acquired using an inverted Leica DM4000B microscope and analysed using Leica application suite software. The number and type of amyloid plaques in the hippocampus were manually defined and quantified. Dense plaques were identified by the presence of dense core whereas diffuse plaques were identified by the presence of diffuse corona, as illustrated in Fig. 3d.

### Neurosphere cultures

Mice were handled in compliance with the relevant national and international guidelines following protocols approved by the Animal Care Ethic Committee of the Biodonostia Institute. Nine-months old 5XFAD and 5XFAD/p38α∆-N mice were euthanized by CO_2_ inhalation and brains were excised and placed in ice-cold PBS (n = four per genotype). The tissue containing the DG was carefully dissected out and homogenized in DMEM-F12 medium containing 10% papain (Roche, 10108014001) and 1% DNAse (Promega, M6101), passed through a 40 μM filter to obtain a single cell suspension, and plated in triplicates in six well culture dishes at a density of 10^5^ cells/well in DMEM-F12 complete culture media (Fisher, 11514436) supplemented with 1% glutamine (Teknovas, A1125030024), 100 units/ml penicillin and 100 μg/ml streptomycin (Teknovas, A3915140122), 1.37% glucose (Sigma, G8769), 1% N2 (Teknovas, 11520536), 2% B27 (Teknovas, 11500446), 20 ng/ml EGF (Gibco, A15E9644), and 20 ng/mL bFGF (Gibco, A15F0291). At day 10, primary neurospheres were counted. Then cells were disaggregated with accutase (Fisher, A1110501) and cultured for additional 10 days before counting secondary neurospheres.

### Statistical analysis

Results are expressed as mean ± SEM of the indicated number of independent experiments. For statistical comparisons among groups, *p* values were calculated using Student’s *t*‐test or with one-way ANOVA followed by Bonferroni’s multiple comparison test. Differences were considered statistically significant when *p* < 0.05 (*p < 0.05; **p < 0.01; ***p < 0.001).

## Additional Information

**How to cite this article**: Colié, S. *et al*. Neuronal p38α mediates synaptic and cognitive dysfunction in an Alzheimer's mouse model by controlling β-amyloid production. *Sci. Rep.* **7**, 45306; doi: 10.1038/srep45306 (2017).

**Publisher's note:** Springer Nature remains neutral with regard to jurisdictional claims in published maps and institutional affiliations.

## Supplementary Material

Supplementary Information
