# Supplementary material for: Neuronal p38α mediates synaptic and cognitive dysfunction in an Alzheimer’s mouse model by controlling β-amyloid production
Source: Sci Rep. 2017 Mar 31;7:45306. doi: 10.1038/srep45306 (PMC5374488; doi:10.1038/srep45306)
Supplement: Supplementary Information [file srep45306-s1.pdf]

Supplementary Information

Neuronal p38α mediates synaptic and cognitive dysfunction in an Alzheimer's mouse model by controlling β-amyloid production

Sandra Colié, Sara Sarroca, Rocío Palenzuela, Idoia Garcia, Ander Matheu, Rubén Corpas, Carlos G. Dotti, José A. Esteban, Coral Sanfeliu and Angel R. Nebreda

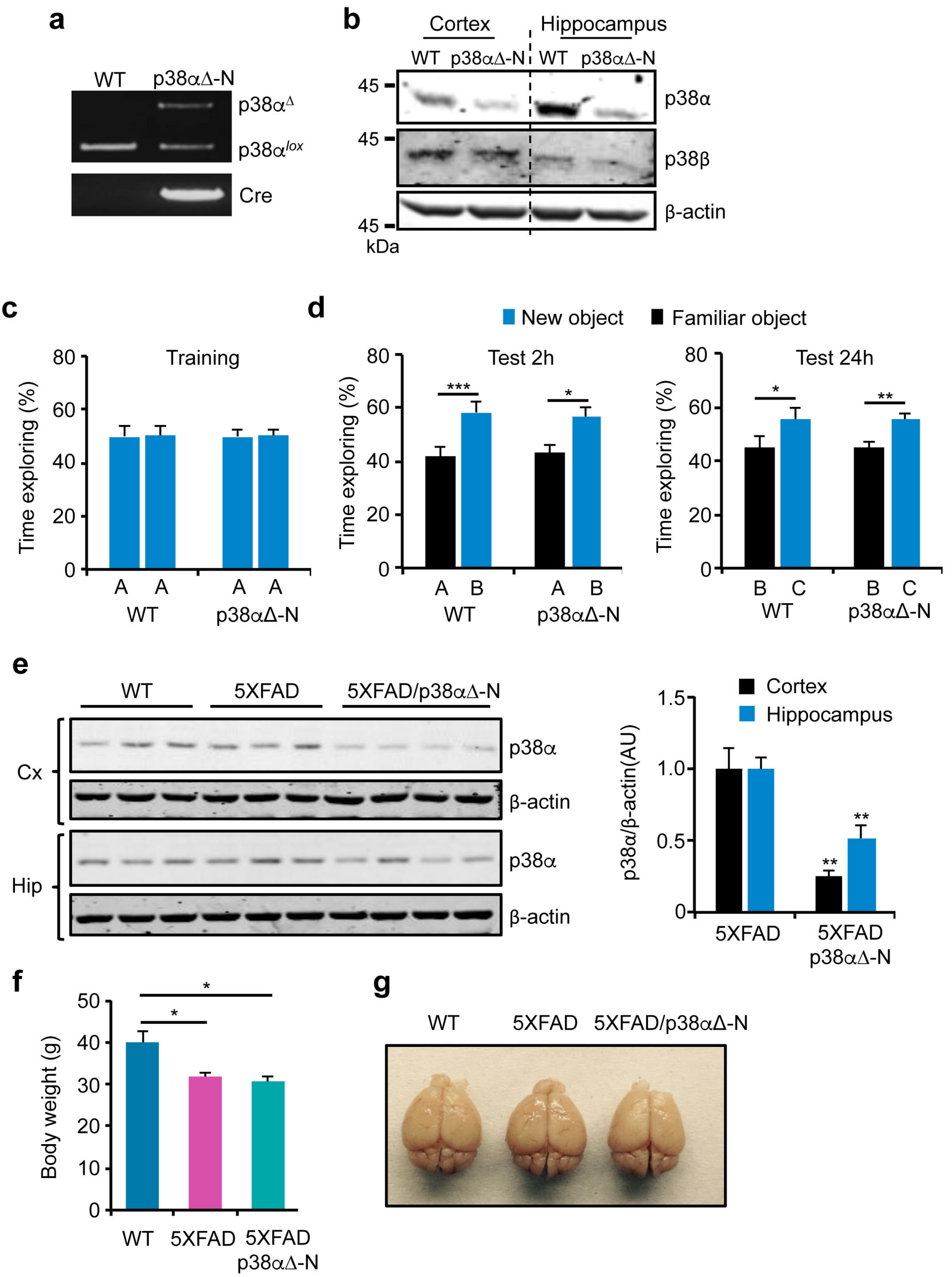

Supplementary Figure S1. Downregulation of neuronal p38α. (**a**) Genomic DNA was extracted from cortical tissue of 3 month old mice and amplified using primers specific for the alleles p38α ^{lox} allele (188-pb) and p38α$^{\Delta}$(411-pb), as well as for Cre (520-pb). (**b**) Western

blot analysis of p38α and p38β expression in the cortex and hippocampus of 3 month old mice either WT or with p38α downregulation in neurons (p38αΔ-N). (**c** and **d**) 4-5-month old mice were analysed using the novel object recognition test. In the training phase, animals were exposed to two identical objects (A and A') for 10 min. After 2 h the A' object was replaced by a new object B (test 2 h) for 10 min. After 24 h, the A object was replaced by a new object C (test 24 h) for 10min. The percentage of time that the mice spent exploring the novel and familiar object over the total time spent exploring both objects were calculated in the training phase (**c**), and test 2 h or test 24 h (**d**). Results are expressed as mean ± s.e.m. ($n \geq 7$). Student *t*-test. ** $p < 0.01$; * $p < 0.05$. (**e**) Western blot analysis of p38α expression in the cortex and hippocampus from 4-5 month old WT, 5XFAD and 5XFAD p38αΔ-N mice. The histrogram shows the quantification of p38α in the Western blots. Results are shown as mean ± s.e.m. ($n \geq 6$). Student *t*-test. ** $p < 0.01$. (**f**) Body weight was recorded at 12 months of age. Results are shown as mean ± s.e.m. ($n \geq 6$). (**g**) Representative pictures of brains from WT, 5XFAD and 5XFAD p38αΔ-N mice.

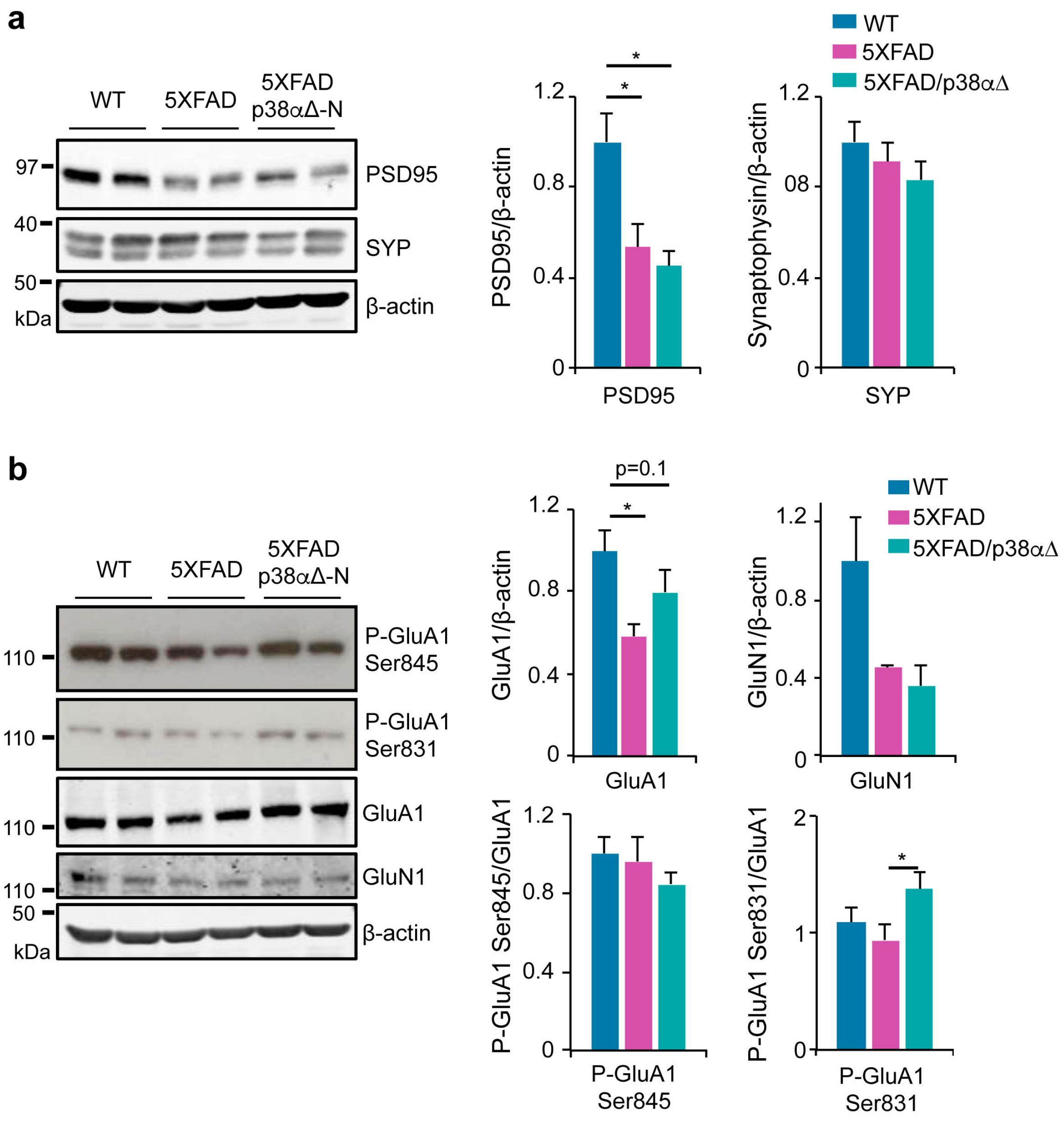

Supplementary Figure S2. Effect of neuronal p38α downregulation on the expression of synaptic proteins and glutamate receptors in 5XFAD mice. Hippocampus lysates from 12 month old mice were analysed by SDS-PAGE and western blotting using antibodies for the postsynaptic density 95 protein (PSD95) and the presynaptic marker synaptophysin (SYP) (**a**), or the glutamate receptors GluA1 and GluN1, and GluA1 phosphorylated Ser845 and Ser831(**b**). The histograms show quantifications normalized to β-actin, in the case of PSD95, SYP, GluA1 and GluN1, and to GluA1 in the case of phospho-antibodies. Results are expressed as mean ± s.e.m. (n≥4). Student *t*-test. * $p < 0.05$.

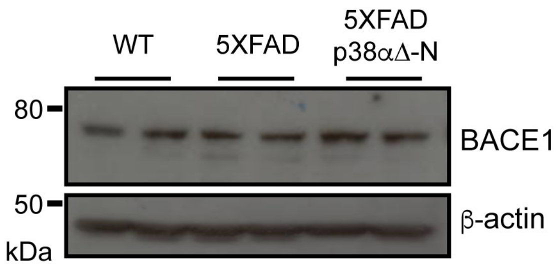

Supplementary Figure S3. Effect of neuronal p38α downregulation on the expression of BACE1 in 5XFAD mice.

Hippocampus lysates from 12 month old mice were analysed by SDS-PAGE and western blotting using antibodies for BACE1

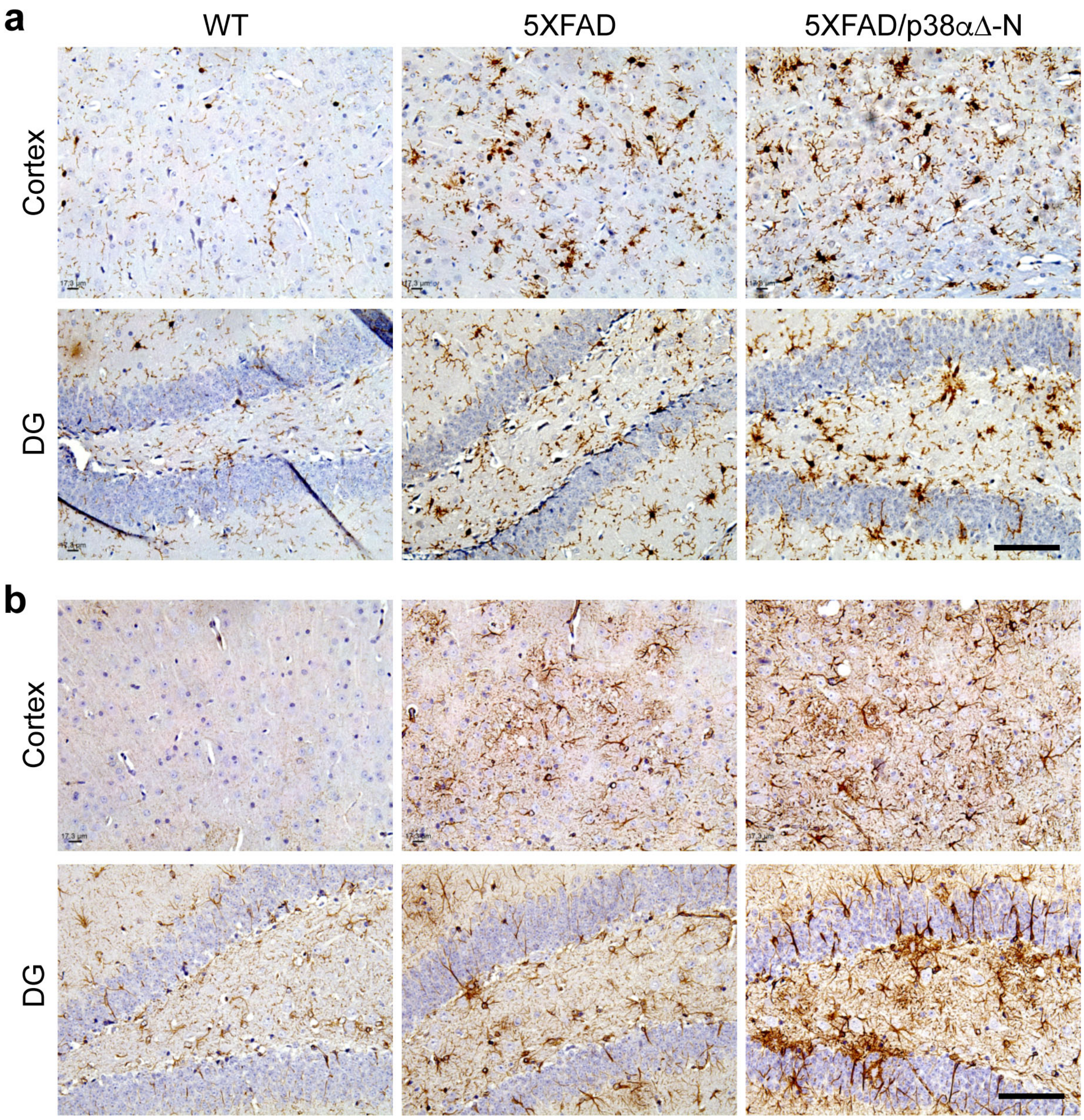

Supplementary Figure S4. Downregulation of neuronal p38α increases astrogliosis in the brain of 5 month old 5XFAD mice. Representative pictures of brain sections from 5-6-month old mice immunostained with antibodies for the migroglia marker Iba1 (**a**) or the astrocyte marker GFAP (**b**). Scale bar = 100 μm.

Supplementary Table S1. Downregulation of neuronal p38α does not alter sensorimotor performance in 5XFAD mice.

	**5XFAD**		**5XFAD/p38αΔ-N**	
	Males<br>n = 6	Females<br>n = 7	Males<br>n = 4	Females<br>n = 9
Body weight (g)	25.20 ± 0.47	20.75 ± 0.39	26.40 ± 1.08	20.21 ± 0.37
Wooden squared rod test (cm)	7.92 ± 2.84	11.79 ± 5.40	15.00 ± 10.21	13.33 ± 4.33
Metal circular rod test (cm)	7.08 ± 4.05	11.07 ± 5.26	3.75 ± 1.61	13.89 ± 4.45
Wire hang test (s)	15.83 ± 3.27	15.00 ± 6.09	13.75 ± 3.15	11.67 ± 3.12
Walking speed (cm/s)	8.26 ± 0.68	6.04 ± 0.27	8.09 ± 1.59	5.93 ± 0.82

Sensorimotor tests are described in García-Mesa et al. (J. Alzheimers Dis. 2011, 24: 421-454). Briefly, in sequential tests, 4-6 month old mice are allowed to walk along a wooden square rod for 20 s, a metal circular rod for 20 s and clung on a wire for 60 s; next day mice freely move in an open field arena for 5 min and walking speed is calculated from a video-recorded track. Results are expressed as mean ± s.e.m. Statistics: no significant difference between 5XFAD and 5XFAD/p38αΔ-N of the same sex (Student's *t*-test).

Supplementary Table S2. Primers used for RT-PCR

Primer	Sequence
BACE-F	5′-aggcagtctctggtatacacccatc
BACE-R	5′-tgccactgtccacaatgctc
TNFα-F	5'-tcatgcaccaccatcaagga
TNFα-R	5′-gaggcaacctgaccactctcc
CXCL12-F	5'-gcagactgtgttgggtgaga
CXCL12-R	5'-catctatcctccccacgaga
rpL32-F	5′-tgtcctctaagaaccgaaaagc
rpL32-R	5′-cgttgggattggtgactctga

Supplementary Table S3. Antibodies used for Western blotting

Antibody	Dilution WB	Brand	Reference
β-actin	1/15000	Abcam	ab49846
ADAM10	1/1000	Abcam	ab1997
β-amyloid (4G8)	1/1000	Covance	SIG-39220
β-amyloid (6E10)	1/1000	Covance	SIG-39320
BACE (M83)	1/200	Santa Cruz	sc-10748
GluA1	1/1000	Abcam	Ab31232
IDE	1/1000	Calbiochem	97-273
GluN1	1/500	Millipore	Ab9864
P-GluA1 ser831	1/500	Millipore	Ab5847
P-GluA1 ser845	1/1000	Affinity Bioreagents	OPA1-04118
Neprilysin	1/300	Abcam	ab951
P-p38	1/500	Cell Sign Technology	9211
p38α	1/500	Cell Sign Technology	9218
p38β	1/200	Invitrogen	33-8700
PSD95	1/500	Millipore	MAB1598
Synaptophysin	1/500	Millipore	MAB368
Tubulinα	1/10000	Sigma Aldrich	T9026
